# Impact of Diet Consistency on the Mandibular Morphology: A Systematic Review of Studies on Rat Models

**DOI:** 10.3390/ijerph19052706

**Published:** 2022-02-25

**Authors:** Ioanna I. Karamani, Ioannis A. Tsolakis, Miltiadis A. Makrygiannakis, Maria Georgaki, Apostolos I. Tsolakis

**Affiliations:** 1 Department of Orthodontics, School of Dentistry, National and Kapodistrian University of Athens, 11527 Athens, Greece; mimak90@hotmail.com (M.A.M.); atsolakis@dent.uoa.gr (A.I.T.); 2 Department of Orthodontics, School of Dentistry, Aristotle University of Thessaloniki, 54124 Thessaloniki, Greece; tsolakisioannis@gmail.com; 3 Department of Oral Pathology and Hospital Dentistry, School of Dentistry, National and Kapodistrian University of Athens, 11527 Athens, Greece; mar1georgaki@gmail.com; 4 Department of Orthodontics, School of Dental Medicine, Case Western Reserve University, Cleveland, OH 44106, USA

**Keywords:** rat, mandible, food consistency, anatomy, growth

## Abstract

Apart from genetics, environmental factors, such as food consistency, may affect craniofacial morphology and development. The present systematic review aims to systematically investigate and appraise the available evidence regarding the effect of diet consistency on the anatomical structures of the basal bone of the rat mandible. The search was performed without restrictions in five databases (PubMed, Scopus, Web of Science, ProQuest Dissertations and Theses Global, including grey literature) and hand searching through January 2022. A total of 14,904 references were initially identified, and 16 articles were finally included in the systematic review. Rats that consumed hard diets were found to exhibit an increase inbigonial width, corpus height, condylar depth, condylar base inclination, condylar process inclination, mandibular plane inclination, height and length of angular process, mandibular body height, depth of antegonial notch, growth rate in the gonial angle, angular process convexity and height of condylar process. It was also noted that mandibular depth, mandibular height, ramus angle and angle between the angular process and mandibular plane were decreased in rats that were fed with a hard diet. On the other hand, there were conflicting results about the growth of mandibular length and width, corpus length, mandibular body length, ramus height, condylar length and width, gonial angle and height of coronoid process. From the abovementioned results, it can be concluded that food consistency may affect the morphology of anatomical structures and the overall growth and development of rat mandibles in various ways.

## 1. Introduction

It is well-established that genetics has a great impact on the development of craniofacial morphology and occlusion—or malocclusion. The study of craniosynostosis has concluded that gene mutations, such as those concerning glupican 1 (GPC1) and gluypican 3 (GPC3), are likely to mediate changes to mandibular size [1]. Moreover, local and systemic inflammation affects the craniofacial morphology, including the mandible and the alveolar bone. Osteoclasts and osteoblasts are the main factors responsible for the dynamic equilibrium between bone resorption and formation in the craniofacial complex. Apart from these two vital components, a complex cellular communication network, including osteocytes, macrophages, monocytes, neutrophils and adaptive immune cells, also plays a crucial role in maintaining strict bone homeostasis [2]. Finally, experiments in animals and studies in humans have implicated pro-inflammatory cytokines (e.g., interleukin-1, tumor necrosis factor alpha and interleukin-6) as paramount mediators of physiologic and pathological bone remodeling [3].

Furthermore, enzyme activity of the masticatory muscles is affected by food hardness due to differences in mechanical stress. It has been shown that changes in diet hardness affect muscular fiber phenotype and enzyme activity encoded by the nuclear and the mitochondrial genome. Moreover, differences in mRNA expression and expression levels of the myosin heavy chain isoform genes MYH 1 and 2 were revealed in experimental rats fed with a soft or hard diet [4]. Many genes were found to be significantly differentially expressed between hard- and soft-diet-fed mice in groups of the same feeding period. The expression of several genes involved in the regulation of actin cytoskeleton was down regulated in the soft-diet-fed mouse [5]. Furthermore, hard diet causes changes in oxidative metabolism and in mRNA levels for the ND1 and 75 kDa MyHC isoforms IIa and IId/x in the superficial portion of rat masseter muscle, but no changes have been found in the composition of muscle fiber types [6].

Families and generations have been observed presenting similarities in their craniofacial and occlusal patterns, and it is accepted that the type of occlusion and the craniofacial growth are heritable to some degree [7,8,9]. However, it has been observed that many civilized human populations have developed more severe malocclusions compared to populations under primitive conditions of life [10]. During that era, an excellent function of jaws and a well-developed masticatory system were essential to chew raw or partially cooked meat in order to survive [11]. In addition, a lower prevalence of severe malocclusions in populations living in rural villages compared with those living in urban industrialized societies has been noted [12,13]. A plausible explanation for these variations is the difference in food consistency of modern urban diets. Consequently, apart from the possible genetic and inflammatory trends and factors, environmental conditions also play a significant role. The effect of food consistency has been studied in various animal species, such as rats, ferrets, mice, pigs, bats, snakes and primates [14,15,16,17,18,19,20,21].

Amongst them, rats are the most frequently used animals. The majority of experimental studies include rats because they are small in size, can be easily housed and reproduced, report a minimal social concern, have a short lifespan and their genetic background and growth are well known and resemble those of humans [22].

A systematic review by Scheidegger et al. (2018) examined the impact of dietary consistency on temporomandibular joint/condyle, condylar cartilage, alveolar bone and periodontal ligament [23]. Therefore, a decision was made to conduct a systematic review that would investigate the effect of diet consistency on the mandible by assessing the morphology of its anatomical structures located away from teeth and alveolus of rats and to appraise the relevant evidence. The only common topic of our study with the aforementioned systematic review will be the condylar dimensions.

## 2. Materials and Methods

### 2.1. Protocol and Registration

A specific protocol was developed and piloted following the guidelines outlined in the PRISMA-P statement [24]. The protocol was subsequently registered in PROSPERO (CRD42019136315) and formed the basis for this review. The Cochrane Handbook for Systematic Reviews of Interventions [25] and the PRISMA statement [26] were followed during conduct and reporting.

### 2.2. Eligibility Criteria

The participants, intervention, comparison, outcomes and study design (PICOS) acronym were applied to define the eligibility criteria (Table S1).

Studies involving rats which were divided into a hard-food-diet group and a soft-food-diet group were reviewed. The effect of food consistency on morphological anatomy of the mandible of rats should be assessed after the measurement of differences in mandibular anatomical structures after at least 1 month in rats consuming hard or ordinary diet and rats being fed with soft diet. Non comparative studies, reviews, systematic reviews, and meta-analyses were excluded.

### 2.3. Information Sources and Search Strategy

In total, five databases (including grey literature) were searched from inception to 1 January 2022. One author (M.A.M.) developed detailed search strategies for each database. They were based on the strategy developed for MEDLINE but revised appropriately to take into account the differences in controlled vocabulary and syntax (Table S2).

No restriction was placed on language, date or status of publication. In addition, efforts to obtain additional studies were made by reviewing the reference lists of all eligible studies, excluded studies and other published systematic reviews. The authors of studies were to be contacted to provide additional data, if needed.

### 2.4. Study Selection

The first and second authors (I.I.K. and I.A.T.) electronically assessed the titles and the abstracts of the retrieved records for inclusion independently and in duplicate. They were not blinded to the identity of the authors, their institution or the results of the research. Subsequently, they obtained and assessed using an identical methodology the total report of records considered by either reviewer to satisfy the inclusion criteria.

Disagreements were resolved by discussion or consultation with the fifth author (A.I.T.). A record of all decisions on study identification was kept. As recommended, kappa statistics were not calculated to describe the extent to which assessments by the two authors were the same [25].

### 2.5. Data Collection and Data Items

The same two authors performed data extraction independently, and any disagreements were again resolved by discussion or consultation with the fifth author. Predetermined and pre-piloted data collection forms were used to record the following information: bibliographic details of the study, details on study design and verification of study eligibility, characteristics of the subjects and the type of food in each group, details on the intervention, outcome measurement characteristics and results.

### 2.6. Risk of Bias in Individual Studies

The risk of bias in individual studies was assessed by I.I.K. and M.A.M. independently and in duplicate with the SYRCLE’s risk of bias tool [27]. Any disagreements were resolved by discussion or consultation with the fifth author (A.I.T.).

### 2.7. Summary Measures and Synthesis of Results

If deemed possible, the random effects method for meta-analysis was to be used to combine data on craniofacial morphology [28,29]. However, quantitative data synthesis was not carried out as planned because of the lack of an adequate amount of data regarding each of the investigated anatomical structures, as well as differences in the used methods [25].

## 3. Results

### 3.1. Study Selection

**Figure 1:**
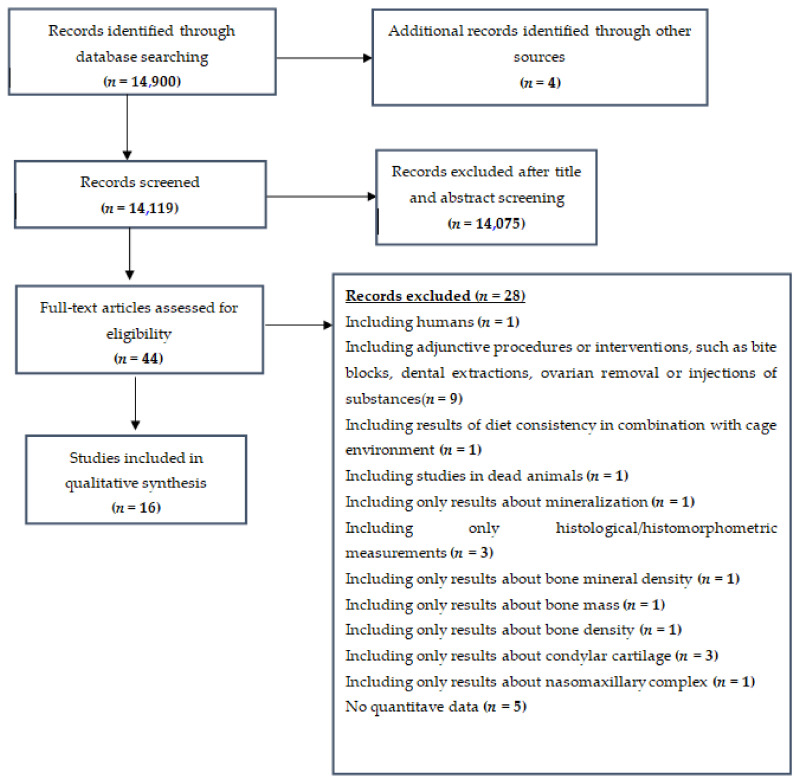
Flowchart—Detailing inclusion/exclusion of studies.

The flow of records through the reviewing process is shown in Figure 1. 

We initially identified 14,904 references and, after the duplicate check, excluded 14,075 more on the basis of their title and abstract. From the 44 records that remained and were assessed for eligibility, 28 studies were excluded for the following reasons: involving human subjects (*n =* 1), including adjunctive procedures, such as bite blocks (*n =* 9), using already dead animals (*n =* 1), having only results related to mineralization of bones (*n =* 1), employing only histological/histomorphometric measurements (*n =* 3), having results of diet consistency related to cage environment (*n =* 1), having results only about bone mineral density (*n =* 1), using results only about bone mass (*n =* 1), having results only about bone density (*n =* 1), involving results only about condylar cartilage (*n =* 3), having results related to nasomaxillary complex only (*n =* 1), having a lack of quantitative data on mandibular morphology (*n =* 5). Finally, 16 full-text study reports were included in the systematic review [30,31,32,33,34,35,36,37,38,39,40,41,42,43,44,45].

### 3.2. Study Characteristics

The characteristics of the included studies are presented in Table 1.

The length of the experimental period varied from 1 month to 7 months. Although the effect of food consistency experiments was mainly conducted on male rats, a few studies used both male and female rats. The majority of studies included young rats in the 21- to 30-day age range, but three studies included both young in the age range of 21 to 28 days and adult rats of 6 weeks to 4 months old. The hard diet group was usually fed with the conventional diet in a hard pellet form and the soft diet group was fed with the same diet but in powdered form mixed with water. The effect on craniofacial morphology was usually assessed by using radiography, morphometric analyses and gross morphological measurements. In a few studies, macroscopic measurements, direct analyses and linear measurements were performed.

### 3.3. Risk of Bias within Studies

Table 2 presents the summary findings of the risk-of-bias assessment.

In general, most studies were considered to present an unclear risk of bias regarding the domains of allocation concealment, random housing of rats during the experiment and assessor blinding, as pertinent details were not clear in most of the papers. Regarding the sequence generation, the risk of bias was rated as unclear for half of the studies and high for the rest because the allocation sequence was either randomized without any further details or not mentioned. The majority of studies presented unclear information on whether the groups of rats were similar at baseline in respect to sex, age and weight. In addition, there was unclear data on whether the caregivers and investigators were blinded, whether animals were randomly selected for outcome assessment, as well as whether incomplete outcome data were adequately addressed. Selective outcome reporting did not appear to be an issue in the retrieved studies. Finally, the information included in the articles was not sufficient to determine the presence of any additional problems that could increase the risk of bias.

### 3.4. Results of Individual Studies

The results of the included studies are presented in Table 1.

The X-ray findings as per the cephalometric analysis showed that the anterior corpus length [30], the ramus height [30,36,41], the bigonial width [30], the growth rate in the gonial angle of mandible [37], the angular process convexity [37], the antegonial notch depth [37], the linear measurements of mandible (the size of the occipital condyle region and the size of the curvature of the angular process) [37] and the height of the condylar process [41] were increased in the hard diet groups. The gonial angle [36], the ramus angle [36] and the angle between angular process and mandibular plane [39] were decreased in the hard diet groups. There was not any significant difference between hard diet and soft diet groups in the mandibular length [36,40,41], the mandibular body length [41], the mandibular base length [36], the length of horizontal branch [40], while there is controversy about the height of coronoid process [36,41].

The gross morphological measurements demonstrated an increase of condylar length [32,33,34], condylar width [32,33,34], condylar depth [33] and mandibular width of weanling rats [33] in hard diet rats. The mandibular length [32,33,34], the mandibular depth [33], the mandibular width of juvenile rats [33] and the ramus height [33] did not differ between hard diet and soft diet rats. In three studies there was also a group which was initially fed with soft food and later with hard food. In these studies, it was found that the condylar depth was larger in hard-diet juvenile rats in relation to soft/hard diet juvenile rats [33], the condylar depth was smaller in hard-diet weanling rats in relation to soft/hard diet weanling rats [33], while there is controversy about condylar length and condylar width between the hard-diet rats and soft/hard diet rats [32,33,34].

The macroscopic measurements showed that the ramus height, the mandibular body length and height and the mandibular base depth were increased in groups with hard diet, while the growth of mandibular length, mandibular ramus curvature depth, mandibular head and gonial angle were the same between hard-diet and soft-diet groups [35].

The planimetric analysis of Barber et al. [44] showed that the condylar area was larger in hard-diet rats, while the condylar length and the mandibular length were similar in both groups.

Through direct and linear measurements, it was found that the length of angular process [42], the mandibular length [31,42], the ramus height I [45] and corpus height [45] were increased in hard-diet groups, while the length of body [42], the condylar length [31], the ramus height II [45] and the sagittal and transversal mandibular measurements [45] did not differ between the two groups. In the study of Beecher, there was also an experimental group referred to as the intermittent group. It was found that the mandibular length was smaller in this group in relation to the hard-diet group, while no difference was observed in the condylar length [31].

The morphometric analyses of Kiliaridis et al. [39] and Ödman et al. [43] showed that the mandibular length and width were increased in the hard-diet group [39]. In addition, it was found that the lateral surface of mandible, the condylar process inclination, the mandibular plane inclination, the condylar base inclination, the posterior height of the corpus and the height of the angular process were larger in hard-diet groups [43]. On the contrary, the condylar process area, the condylar process length, the height of the coronoid process and the distance between mental foramen and condyle or coronoid or angular process did not show any difference between hard-diet and soft-diet groups [43].

## 4. Discussion

It was in the 1960s when Melvin Moss developed the functional matrix theory. According to his theory, “growth of the face occurs as a response to functional needs and neurotrophic influences and is mediated by the soft tissue in which the jaws are embedded” [46]. The origin, the development, the changes in shape and size, the location and the maintenance of cranial skeletal elements are always secondary, compensatory and necessary responses to chronologically and operationally prior events or processes that occur in non-skeletal cells, tissues and organs [47]. Later on, Moss revised his theory, stating that function in combination with genetics were both accountable for the procedures of growth and development [48].

Several methods have been used to assess the way function influences the development of craniofacial morphology. The main methods that are presented in the literature are the following: observation of the craniofacial complex either in patients with muscular dystrophy, in animal subjects following the incision or removal of muscles or in animals using the least-invasive feeding with food of different consistencies.

Studies investigating muscular dystrophy indicate that these patients present a craniofacial morphology with vertical aberration, characterized by a large angle between the mandibular and palatal planes and a steep mandible [49]. The maxillary plane angle is larger than normal, while the anterior upper face height is smaller [50]. A reduction of the sagittal skeletal intermaxillary relationship is also observed [51]. In addition to the influence on facial morphology, muscular dystrophy also affects the dental arch dimensions and oral functional capacities. In all patients, a marked transverse increase of the posterior part of the dental arches was found, mostly in the lower one, resulting in posterior crossbites, as well as a tendency towards a skeletal Class III relationship [51].

Likewise, injection of botulinum neurotoxin type A (BoNT/A), which causes masticatory atrophy and paresis in the masseter or temporalis muscle of rats, exhibited a facial morphology typical of a dolichofacial profile: short upper face accompanied by a long lower face with a rather increased mandibular length and ramus height and constricted bicoronoidal and bigonial widths [52]. The unilateral removal of the masseter muscle during the growing period in rats induced atrophic changes in the angular process, as well as an “open bite” on the side of operation, asymmetry of the maxilla and shortening of the whole mandible, which on that side became warped in an inferior and lateral direction [53,54], while the bilateral removal of the masseter muscle resulted in no marked gross or dental changes [54].

As far as dietary consistency is concerned, there has been another systematic review that examines its effect on dentoalveolar bony structures of healthy mice and rats, as well as on the periodontal ligament and the region of the mandibular condyle [23].

The laboratory rat is an indispensable component of today’s biomedical research. Rats are the most-used animals for experimental purposes (accounting for approximately 20% of the total number of mammals used for scientific purposes). They have been included in investigations in most aspects of biomedical, behavioral and nutritional research [55]. In our study, we examined how the alteration of food consistency affects the anatomy of various structures of the mandible of healthy rats.

It was noted that there was an agreement about the growth and development of bigonial width, condylar base inclination, condylar process inclination, condylar depth, corpus height, mandibular depth, mandibular plane inclination, length and height of angular process, ramus angle, mandibular ramus curvature depth, mandibular base depth, mandibular head, mandibular body height, angle between the angular process and mandibular plane, length of the horizontal branch (from the antegonial notch to the distal alveolar margin of the lower incisor), depth of antegonial notch, height and length of the condylar process, distance between the mental foramen and the condylar or coronoid or angular process, sagittal mandibular measurements, transversal mandibular measurements, linear measurements of mandible, lateral surface of the mandible, growth rate in the gonial angle, angular process convexity, condylar process area and condylar area among the studies.

On the other hand, there was controversy about the growth of mandibular length and width, corpus length, mandibular body length, ramus height, condylar length and width, gonial angle and height of coronoid process. These differences among the results of the studies could be interpreted by a plethora of factors. 

The rats that were tested in the included studies were of different ages (adult, young, juvenile and weanling rats) and, as a result, they could have varying growth potential. In older rats, the large hydrated cells are absent altogether, indicating that the capacity for volume increase is lost with age. The period of rapid growth is the first 5 weeks of life, while growth slows between 8 and 16 weeks. This decrease could be a consequence either of a reduced rate of proliferation or an increased rate of cell death. After 28–30 weeks, growth virtually ceases in rats [56]. In addition, phenomena that appear to be identical in their form may be governed by different mechanisms at different ages [57].

Food consistency may constitute a confounding factor since variation in hardness may exist among the included studies. The majority of hard diet groups were usually fed with the conventional diet in a hard pellet form, and the majority of soft diet groups were fed with the same diet, however in a powdered form mixed with water. In a few studies, hard diet groups continued to be fed with the ordinary diet, while soft diet groups were fed either with the hard diet mixed with water or with the powdered form of the hard diet without mixing with water. None of the authors of the included papers elaborated on a method of assessment of the hardness of the food consumed by the rats.

Furthermore, there is a complete absence of power calculation among the studies. Calculation of sample size is one of the most important components of the design of any research, including animal studies. If a smaller sample size is chosen, it could lead to the loss of potential statistical significance [58,59].

In addition, the rats used amongst the various studies were of different strains. Food consistency could affect different strains in varying ways. A strain, in regard to rodents, is a group in which all members are as nearly as possible genetically identical. In rats, this is accomplished through inbreeding. By having this kind of population, it is possible to conduct experiments to determine the roles of genes or conduct experiments that exclude variations in genetics as a factor [60].

Another factor that differed among the studies is the duration of their follow-up periods. The longer the duration of a follow-up period, the greater is the chance for changes in growth to be recorded. Thus, it would be expected to detect statistically significant results more readily in experiments that had longer follow-ups.

Last, but far from least, it is observed that different outcomes derive from unlike methods of outcome assessment with varying validity. Radiography, and especially cephalometry, presents some limitations, such as image magnification and shape distortion, which could affect the measurements [61]. In addition, the outcomes could be affected by the assessor’s errors. In regards to planimetric analysis, there could have been some minimal variation between different observers. Apart from that, this technique does not offer the option to perform volumetric measurements [62]. Moreover, the measurements of morphometrics have the significant limitation that they contain little information about the spatial distribution of shape changes across the organism [63]. Finally, the macroscopic and gross measurements deal with characteristics that are discernible to the unaided senses and are not appropriate for characteristics hidden from the senses, which may be too small to be visible or be obscured by other factors [64].

### 4.1. Strengths and Limitations

The search strategy used was exhaustive, covering manual, electronic and grey literature material up to January 2022. Every effort was made to minimize bias to the full extent possible. Screening, verification of eligibility, data extraction, as well as assessment of the risk of bias and the quality of evidence were performed in duplicate. Disagreements were resolved by discussion or consultation with the fifth coauthor until a final consensus was achieved.

There are also some limitations to this review, mainly associated with the nature and the characteristics of the included studies, such as the study of one specific animal model and the data retrieved during the review process. The included studies had different methods of outcome assessment. Moreover, the rats, which were used in all studies, were of different age, sex and strain. Most studies were assessed to be of unclear or high risk of bias because of methodological characteristics. Furthermore, it has to be acknowledged that the data retrieved in this systematic review have been extracted from animal studies and cannot be directly extrapolated to humans. This is further complicated by the fact that animals, and especially rats, present a similar (mammalian), but not identical, craniofacial morphology as humans and also have different masticatory forces and function of their stomatognathic system.

### 4.2. Recommendations for Future Research

As the potential effect of diet consistency on the anatomy of the mandible is very useful and significant information, further research is warranted. However, it is clear that such experiments have ethical limitations (i.e., they would never be performed on human beings). Thus, animal studies seem to be the only way to further investigate this topic, at least for the time being. The case of alteration of craniofacial morphology due to food consistency potentially causing alterations in the genetic material to be inherited by the offspring warrants studies that would follow consecutive generations of rats. Moreover, a method of assessment of the hardness of the food consumed by the animals would be very useful to be included, as the level of mechanical load which is applied to bones through muscular function by hard diet could be an important factor for growth and development of the craniofacial region. Finally, study designs should receive the necessary standardization and generalization, and possible sources of bias should be controlled.

## 5. Conclusions

Diet consistency may exhibit various effects on the morphology of a rat’s mandible. However, these results should be seen with caution. It should not be ignored that the data in this systematic review have been extracted from animal studies and cannot be directly extrapolated to humans, as craniofacial morphology and function differ between animals and humans.

## Figures and Tables

**Table 1:** Characteristics of the studies included in the systematic review.

Authors; Year	Subject Characteristics (Species; Sample Size; Gender; Age; Weight)	Hard Diet Group (Number, Type of Food, Treatment Duration)	Soft Diet Group (Number, Type of Food, Treatment Duration)	Frequency of Outcome Measurements	Method of Outcome Assessment	Results
**Abed et al., 2007** [30]	wild-type Sprague–Dawley rats; 32; M; 23 d; not mentioned	N = 16 hard pellets until the age of 79 d	N = 16 grinding the hard diet pellets to a powder in a blender until the age of 79 d	T1 = 23 d of age T2 = 37 d of age T3 = 51 d of age T4 = 65 d of age T5 = 79 d of age	(a) lateral cephalograms (b) dorso–ventral cephalograms The computer software used for digitization and measurement was Viewbox1	**Size differences** **anterior corpus length:** HD > SD **ramus height:** HD > SD **Differences in growth velocity** **anterior corpus length:** HD > SD **ramus height:** HD > SD **Differences in growth deceleration** **bigonial width:** HD > SD
**Barber et al., 1963** [44]	Long–Evans rats; 30; M; 21 d; not mentioned	N = 15 whole pellets 18 wks	N = 15 ground rat diet 18 wks	after 18 wks	Planimetric analysis: Overhead projector was used to project a silhouette of the mandibular condyles. Polar planimeter was employed to measure the area of the condylar tracings. Three readings of area were taken and a mean of each three readings was computed.	**Projected tracing measurements** **condylar area:** HD > SD **condylar length:** n **Actual measurements** **mandibular length:** n **mandibular weight:** HD > SD
**Beecher and Corruccini; 1981** [31]	Sprague–Dawley rats; 45 Mand 45 F; 21 d; not mentioned	Group I: N = 30 pelleted rat chow 4 mos	Group II: N = 30 gruel-like porridge consisting of ground chow moistened with water 4 mos Group III: N = 30 soft diet 6 d with dry pellets every 7th d 4 mos	after 4 mos	Direct anteroposterior length measurement on condyle	**Mandibular length:** HD > S/HD > SD **Condylar length:** n
**Bouvier; 1988** [32]	Sprague–Dawley rats; young rats: 20 Mand 26 F; 21 d; not mentioned adult rats: 31 M; 4 mos; 467 ± 48 g	Group I: N = 28 (16 young and 12 adult) hard, pelleted rat chow young: for 4 wks adult: for 12 wks	Group II: N = 26 (16 young and 10 adults) ground rat chow moistened with water young rats: for 4 wks adult rats: for 12 wks Group III: N = 23 (14 young and 9 adults) moist, ground chow for the first 2 wks (young) or 6 wks (adult) and then for the remaining 2 wks (young) or 6 wks (adult), hard pelleted chow	young rats: 4 wksadult rats: 12 wks	Gross measurements with calipers	**Mandibular length:** n **Condylar length:** young rats (females): HD > SD S/HD > SD HD n S/HD young rats (males): HD > SD S/HD n SD HD n S/HD adult rats (males): HD > SD S/HD > SD HD n S/HD **Condylar width:** young rats (females): HD > SD S/HD > SD HD n S/HD young rats (males): HD > SD S/HD > SD HD n S/HD adult rats (males): HD > SD S/HD > SD HD > S/HD
**Bouvier and Hylander;** **1984** [33]	Long–Evans rats; weanling rats: 15 M; 23 d; not mentioned juvenile rats: 15 M; 6 wks; not mentioned	Group Ia: N = 5 weanlings hard, pelleted rat chow 5 wks Group Ib: N = 5 juveniles hard, pelleted rat chow 8 wks	Group IIa: N = 5 weanlings softened rat chow 5 wks Group IIb: N = 5 juveniles softened rat chow 8 wks Group IIIa: N = 5 weanlings softened rat chow: 2 wks hard, pelleted rat chow: 3 wks Group IIIb: N = 5 juveniles softened rat chow: 4 wks hard, pelleted rat chow: 4 wks	weanlings: after 5 wks juveniles: after 8 wks	Gross morphological analysis: one dentary, the hind limb and the remaining portion of the head were placed in 10% neutral buffered formalin and stored for later gross morphological analysis.	**Weanling rats** **Condylar length:** HD = S/HD > SD **Condylar width:** HD = S/HD > SD **Condylar depth:** HD > SD HD <S/HD S/HD > SD **Mandibular length:** n **Mandibular depth:** n **Mandibular width:** HD > S/HD > SD **Ramus height:** n **Juvenile rats** **Condylar length:** HD > H/SD > SD **Condyle width:** HD > S/HD > SD **Condyle depth:** HD > S/HD > SD **Mandibular length:** n, except F-G area HD > H/SD HD > SD **Mandibular depth:** n **Mandibular width:** n **Ramus height:** n
**Bouvier and Zimny;** **1987** [34]	Sprague–Dawley rats; young rats; 12 M and 26 F; 4 wks; not mentioned mature adult rats: 31 M; not mentioned; not mentioned	Group IaM N = 6 young Ms hard pelleted rat chow 4 wks Group IaF: N = 9 young females hard pelleted rat chow 4 wks Group IIa: N = 14 adults hard pelleted rat chow 12 wks	Group IbM: N = 3 young Ms ground moistened chow 4 wks Group IbF: N = 9 young Fs ground moistened chow 4 wks Group IcM: N = 3 young Ms softened chow 2 wks hard chow 2 wks Group IcF: N = 8 young Fs softened chow 2 wks hard chow 2 wks Group IIb: N = 12 adults ground moistened chow 12 wks Group IIc: N = 9 adults softened chow 6 wks hard chow 6 wks	weanlings: after 4 wks adults: after 12 wks	Gross morphological measurements using dial calipers; Scanning EM sample preparation: the condyle was separated from the mandibular body and samples were processed for scanning electron microscopy	**Young females** **Mandibular length:** n **Condylar length:** HD > S/HD > SD **Condylar width:** HD > S/HD > SD **Young males** **Mandibular length:** n **Condylar length:** n **Condylar width:** n **Old males** **Mandibular length:** n **Condylar length:** HD > S/HD > SD **Condylar width:** HD > S/HD > SD
**Guerreiro et al., 2013** [35]	Wistar rats; 24 M; 21 d; not mentioned	N = 12 a solid diet, laboratory chow for rats in a hard commercial pellet form 50 d	N = 12 a powdered diet, which consisted of ground and sieved commercial pellets 50 d	after 50 d	Macroscopic measurements: Macroscopic measurements were carried out on digital photographs. Some reference landmarks and measurements used in this study were adapted from cephalometric measurements (saggital, vertical, angular)	**Mandibular length:** n **Mandibular body length:** HD > SD **Mandibular ramus curvature depth:** n **Mandibular head:** n **Mandibular base depth:** HD > SD **Ramus height:** HD > SD **Mandibular body height:** HD > SD **Gonial angle:** n
**Hichijo et al., 2014** [36]	Wistar strain rats; 14 M; 3 wks; not mentioned	N = 7 solid hard diet (ordinary pellet) 11 wks	N = 7 powder soft diet (ordinary pellet) 11 wks	after 11 wks	Cephalometric analysis:lateral cephalograms were taken of each right hemimandible using a rat and mouse cephalometer with dental occlusal film. Cephalometric analysis was performed by means of ABS Digimatic Caliperis	**Total length of mandible:** n **Base length of mandible:** n **Height of coronoid process:** n **Mandibular ramus height:** HD > SD **Gonial angle:** HD < SD **Ramus angle:** HD < SD
**Kiliaridis et al., 1999** [39]	Sprague–Dawley rats; 40 M; 28 d; 100 ± 2 g	N = 20 ordinary diet for rats in hard pellet form 28 d	N = 20 ordinary diet, ground and mixed with water in standardized proportions 28 d	after 28 d	Gross morphometric analysis: lateral and axial photos were taken from the dry mandibles of 10 animals in a standardized way by means of a stereo microscope (Olympus)	**Mandibular length:** HD > SD **Mandibular width:** HD > SD
**Kiliaridis et al., 1985** [37]	Sprague–Dawley rats; 32 M; not mentioned; 100 g	N = 16 ordinary diet 28 d	N = 16 ordinary diet mixed with water in standardized proportions 28 d	T1 = beginning T2 = 14 d after T1 T3 = 28 d after T1	Lateral cephalograms; A standard length of wire (10 mm) attached to each film; Computerised cephalometric analysis was performed; The measurements were calibrated according to the image of the standard length of the wire.	**Angular process convexity:** HD > SD (28 d) **Antegonial notch depth:** HD > SD (28 d) **Linear measurements of the mandible** (size of the occipital condyle region, size of the curvature of the angular process): HD > SD **Growth rate at the gonial angle of mandible:** HD > SD
**Kiliaridis; 1986** [38]	Sprague–Dawley rats; 36 M; not mentioned; 90 g	N = 12 ordinary diet of hard pellets 29 d	SD1: N = 12 same ordinary rat diet in a ground form mixed with water 29 d SD2: N = 12 same ordinary rat diet in a ground form mixed with water + incisors shortened regularly by grinding with a diamond disc. No incisal contact was possible for some time after this procedure. 29 d	T1 = beginning (Oxytetracycline injection) T2 = 15 d from T1 (Oxytetracycline injection) T3 = 27 d from T1 (Oxytetracycline injection) T4 = 29 d from T1	lateral cephalograms and computerized cephalometric analysis	**Angle (angular process-mandibular plane):** HD < SD1< SD2 (T4) **Antigonial notch depth:** HD > SD2 SD1 > SD2 (T4)
**Luca et al., 2003** [40]	Sprague–Dawley rats; 30 M; 28 d; not mentioned	N = 10 normal diet (pellets) 28 d	SD1: N = 10 liquid diet (dry powder of pellets dissolved in water) 28 d SD2: N = 10 elastic diet (similar to normal diet, with binders and gums added) 28 d	after 28 d	Cephalometric analyses: lateral radiographs of the cranium	**Posterior mandibular length:** n **Length of the horizontal branch (from the antegonial notch to the distal alveolar margin of the** **lower incisor):** n
**Maki et al., 2002** [41]	Wistar rats; 30 M; 3 wks; 40 g	N = 10 hard diet (CA-1) 6 wks	SD1 (kneaded-diet): N = 10 diet of powder CA-1 that had been first kneaded with water and then left to dry 6 wks SD2 (powdered-diet): N = 10 powdered diet 6 wks	after 6 wks	Lateral cephalometric analysis: the cranial bones were cut laterally along the median suture from the parietal bone to the mandible. The median sagittal face of the left side of the head was mounted to contact the film surface, with the mental foramen set immediately under the focus. Setting of coordinate axes and actual length measurements: An X-axis was constructed parallel to the mandibular plane and a perpendicular Y-axis was constructed through the mental foramen. These 2 lines were set as the X and Y coordinates. A slide caliper was used to measure the length.	**Mandibular length:** n **Mandibular body length:** n **Mandibular ramus height:** HD > SD1, SD2 **Height of the condylar process:** HD and SD1: n HD, SD1 > SD2 **Height of the coronoid process:** HD > SD2
**Moore; 1964** [42]	albino rats from the Birmingham colony; 20 M and 20 F; 3 wks; not mentioned	N = 20 standard rat cake 17 wks	N = 20 ground rat cake mixed with water 17 wks	after 17 wks	Linear dimensions were taken with vernier calipers and recorded to the nearest 0.05 mm. Weights of both bones and muscles were recorded to the nearest 0.001 g.	**Mandible weight:** HD > SD (M and F) **Overall length of mandible:** HD > SD (F) n (M) **Length of body:** n (M and F) **Length of angular process:** HD > SD (M and F)
**Ödman et al., 2008** [43]	Sprague–Dawley rats; 60 M; 21 d; not mentioned	N = 16 ordinary (hard) food 27 wks	N = 44 ordinary (hard) food mixed with water 21 wks SD1: N = 22 continued on soft diet 6 wks SD2: N = 22 changed to an ordinary (hard) diet 6 wks	after 27 wks	Morphometric analysis of the mandibular lateral shape was used: each left mandible was photographed using a digital camera; the images acquired were subsequently transferred to acomputer for interpretation using customized cephalometric software	**Total area of lateral surface of mandible:** HD > SD1 **Condylar process area:** n **Condylar process inclination:** HD > SD1 HD > SD2 **Mandibular plane inclination:** HD > SD2 **Condylar base inclination:** HD > SD1 SD2 > SD1 **Condylar process length:** n **Mental foramen—condyle process:** n **Mental foramen—coronoid process:** n **Mental foramen—angular process:** n **Posterior height of the corpus:** HD > SD1 **Height of coronoid process:** n **Height of angular process:** HD > SD1 HD > SD2
**Ulgen et al., 1997** [45]	Wistar albino rats; 35 M; 30 d; not mentioned	N = 21 hard pellet food 60 d	N = 14 food pellets were comminuted to inhibit the gnawing activity 60 d	at age of 90 d	Direct measurements were made on the adult skulls and mandibles with a compass.	**Sagittal mandibular measurements:** **total mandibular length I (distance between the infradental and the condylion points):** n **total mandibular length II (distance between the infradental point** **and the coronoid point):** n **corpus length:** n **Vertical mandibular measurements** **ramus height I (distance between the condylion point and the gonion tangent point):** HD > SD **ramus height II (distance between the coronoid point and the gonion tangent point):** n **corpus height:** HD > SD **Transversal mandibular measurements:** **bicoronoidal width:** n **bicondylar width:** n **bigonial width:** n

N, number; M, males; F, females; Ms, males; Fs, females; d, days; wks, weeks; mos, months; g, grams; n, no significant difference; HD, hard diet group; SD, soft diet group; H/SD, group fed initially with hard diet and later with soft diet; S/HD, group fed initially with soft diet and later with hard diet.

**Table 2:** Risk of Bias Assessment with SYRCLE.

	Signaling Questions									
	1	2	3	4	5	6	7	8	9	10
**Abed et al.** [30]	Unclear	Unclear	Unclear	Unclear	Unclear	Unclear	Low	Unclear	Low	Unclear
**Barber et al.** [44]	High	Unclear	Unclear	Unclear	Unclear	Unclear	Unclear	Low	Low	Unclear
**Beecher and Corruccini** [31]	High	Unclear	Unclear	Unclear	Unclear	Unclear	Low	Unclear	Low	Unclear
**Bouvier** [32]	High	Unclear	Unclear	Unclear	Unclear	Unclear	Unclear	Low	Low	Unclear
**Bouvier and Hylander** [33]	High	Unclear	Unclear	Unclear	Unclear	Unclear	Unclear	Unclear	Low	Unclear
**Bouvier and Zimny** [34]	High	Unclear	Unclear	Unclear	Unclear	Unclear	Unclear	Unclear	Low	Unclear
**Guerreiro et al.** [35]	Unclear	Unclear	Unclear	Unclear	Unclear	Unclear	Unclear	Unclear	Low	Unclear
**Hichijo et al.** [36]	Unclear	Unclear	Unclear	Low	Unclear	Unclear	Unclear	Unclear	Low	Unclear
**Kiliaridis (1986)** [38]	High	Unclear	Unclear	High	Unclear	Unclear	Unclear	Unclear	Low	Unclear
**Kiliaridis et al. (1985)** [37]	Unclear	Unclear	Unclear	High	Unclear	Unclear	Low	Unclear	Low	Unclear
**Kiliaridis et al. (1999)** [39]	Unclear	Low	Unclear	Unclear	Unclear	Unclear	Unclear	Low	Low	Unclear
**Luca et al.** [40]	Unclear	Unclear	Unclear	Unclear	Unclear	Unclear	Low	Unclear	Low	Unclear
**Maki et al. [41]**	Unclear	Unclear	Unclear	Unclear	Unclear	Unclear	Unclear	Unclear	Low	Unclear
**Moore [42]**	Unclear	Low	Unclear	Unclear	Unclear	Unclear	Unclear	Unclear	Low	Unclear
**Ödman et al. [43]**	High	Unclear	Unclear	Unclear	Unclear	Unclear	Unclear	Unclear	Low	Unclear
**Ulgen et al.** [45]	High	Unclear	Unclear	High	Unclear	Unclear	Unclear	Unclear	Low	Unclear

(1) Was the allocation sequence adequately generated and applied? (2) Were the groups similar at baseline or were they adjusted for confounders in the analysis? (3) Was the allocation adequately concealed? (4) Were the animals randomly housed during the experiment? (5) Were the caregivers and investigators blinded to the intervention that each animal received? (6) Were animals selected at random for outcome assessment? (7) Was the outcome assessor blinded? (8) Were incomplete outcome data adequately addressed? (9) Are reports of the study free of selective outcome reporting? (10) Was the study apparently free of other problems that could result in a high risk of bias?

## Supplementary Materials

The following are available online at https://www.mdpi.com/article/10.3390/ijerph19052706/s1, Table S1: Eligibility criteria for the present systematic review. Table S2: Strategy for database search (up to January 2022).

## References

[1] Mian M., Ranjitkar S., Townsend G.C., Anderson P. (2017). Alterations in mandibular morphology associated with glypican 1 and glypican 3 gene mutations. Orthod. Craniofac. Res..

[2] Goldring S.R. (2003). Inflammatory Mediators as Essential Elements in Bone Remodeling. Calcif. Tissue Res..

[3] Bonar S.L., Brydges S.D., Mueller J.L., McGeough M.D., Pena C., Chen D., Grimston S.K., Hickman-Brecks C.L., Ravindran S., McAlinden A. (2012). Constitutively Activated NLRP3 Inflammasome Causes Inflammation and Abnormal Skeletal Development in Mice. PLoS ONE.

[4] Ödman A.M., Hunt N.P., Moawad H.A.M., Sinanan A.C.M., Kiliaridis S., Lewis M.P. (2013). Molecular changes in detrained & retrained adult jaw muscle. Eur. J. Orthod..

[5] Seki M., Haino A., Ishikawa T., Inagawa H., Soma G.-I., Terada H., Nashimoto M. (2020). Mastication Affects Transcriptomes of Mouse Microglia. Anticancer Res..

[6] Ide Y., Sato I. (2006). Effect of Changes in Food Consistency on NADH-Ubiquinone Oxidoreductase Activity and Levels of mRNA for ND1, 51 kDa, 75 kDa and Myosin Heavy Chain Isoforms in Two Different Portions of Rat Masseter Muscle. Okajimas Folia Anat. Jpn..

[7] Lundstrom A. (1948). Tooth Size and Occlusion in Twins.

[8] Kraus B.S., Wise W.J., Frei R.H. (1959). Heredity and the craniofacial complex. Am. J. Orthod..

[9] King W.W., Russell S.P., Suckow M.A., Weisbroth S.H., Franklin C.L. (2006). Metabolic, Traumatic, and Miscellaneous Diseases. The Laboratory Rat.

[10] Hunt E.E. (1961). Malocclusion and civilization. Am. J. Orthod. Dentofac. Orthop..

[11] Proffit W.R., Fields H.W., Sarver D.M. (2012). Malocclusion and dentofacial deformity in contemporary society. Contemporary Orthodontics.

[12] Corruccini R.S. (1984). An epidemiologic transition in dental occlusion in world populations. Am. J. Orthod..

[13] Lombardi A.V., Bailit H.L. (1972). Malocclusion in the Kwaio, a Melanesian group on Malaita, Solomon Islands. Am. J. Phys. Anthr..

[14] He T. (2004). Craniofacial morphology and growth in the ferret: Effects from alteration of masticatory function. Swed. Dent. J. Suppl..

[15] Ito G., Mitani S., Kim J.H. (1988). Effect of soft diets on craniofacial growth in mice. Anat. Anzeiger..

[16] Beecher R.M., Corruccini R.S., Freeman M. (1983). Craniofacial correlates of dietary consistency in a nonhuman primate. J. Craniofac. Genet. Dev. Biol..

[17] Hampton P.M. (2011). Comparison of cranial form and function in association with diet in natricine snakes. J. Morphol..

[18] Larsson E., Øgaard B., Lindsten R., Holmgren N., Brattberg M., Brattberg L. (2005). Craniofacial and dentofacial development in pigs fed soft and hard diets. Am. J. Orthod. Dentofac. Orthop..

[19] He T., Kiliaridis S. (2003). Effects of masticatory muscle function on craniofacial morphology in growing ferrets (Mustela putorius furo). Eur. J. Oral Sci..

[20] Burn A.K., Herring S.W., Hubbard R., Zink K., Rafferty K., Lieberman E.D. (2010). Dietary consistency and the midline sutures in growing pigs. Orthod. Craniofac. Res..

[21] Santana S.E., Grosse I.R., Dumont E.R. (2012). Dietary hardness, loading behavior, and the evolution of skull form in bats. Evolution.

[22] Gomes P.S., Fernandes M.H. (2011). Rodent models in bone-related research: The relevance of calvarial defects in the assessment of bone regeneration strategies. Lab. Anim..

[23] Scheidegger R., Koletsi D., Eliades T. (2018). The impact of dietary consistency on structural craniofacial components: Temporomandibular joint/condyle, condylar cartilage, alveolar bone and periodontal ligament. A systematic review and meta-analysis in experimental in vivo research. Arch. Oral Biol..

[24] Shamseer L., Moher D., Clarke M., Ghersi D., Liberati A., Petticrew M., Shekelle P., Stewart L.A., PRISMA-P Group (2015). Preferred reporting items for systematic review and meta-analysis protocols (PRISMA-P) 2015: Elaboration and explanation. BMJ.

[25] Higgins J.P.T., Green S. (2011). Cochrane Handbook for Systematic Reviews of Interventions Version 5.1.0.

[26] Liberati A., Altman D.G., Tetzlaff J., Mulrow C., Gøtzsche P.C., Ioannidis J.P.A., Clarke M., Devereaux P.J., Kleijnen J., Moher D. (2009). The PRISMA statement for reporting systematic reviews and meta-analyses of studies that evaluate health care interventions: Explanation and elaboration. PLoS Med..

[27] Hooijmans C.R., Rovers M.M., de Vries R.B., Leenaars M., Ritskes-Hoitinga M., Langendam M.W. (2014). SYRCLE’s risk of bias tool for animal studies. BMC Med. Res. Methodol..

[28] Borenstein M., Hedges L.V., Higgins J.P.T., Rothstein H.R. (2009). Introduction to Meta-Analysis.

[29] DerSimonian R., Laird N. (1986). Meta-analysis in clinical trials. Control Clin. Trials.

[30] Abed G.S., Buschang P.H., Taylor R., Hinton R.J. (2007). Maturational and functional related differences in rat craniofacial growth. Arch. Oral Biol..

[31] Beecher R.M., Corruccini R.S. (1981). Effects of dietary consistency on craniofacial and occlusal development in the rat. Angle Orthod..

[32] Bouvier M. (1988). Effects of Age on the Ability of the Rat Temporomandibular Joint to Respond to Changing Functional Demands. J. Dent. Res..

[33] Bouvier M., Hylander W.L. (1984). The effect of dietary consistency on gross and histologic morphology in the craniofacial region of young rats. Am. J. Anat..

[34] Bouvier M., Zimny M.L. (1987). Effects of mechanical loads on surface morphology of the condylar cartilage of the mandible in rats. Acta Anat..

[35] Guerreiro F.D.S., Diniz P., Carvalho P.E.G., Ferreira E.C., Avancini S.R.P., Ferreira-Santos R.I. (2013). Effects of masticatory hypofunction on mandibular morphology, mineral density and basal bone area. Braz. J. Oral Sci..

[36] Hichijo N., Kawai N., Mori H., Sano R., Ohnuki Y., Okumura S., Langenbach G.E.J., Tanaka E. (2014). Effects of the masticatory demand on the rat mandibular development. J. Oral Rehabil..

[37] Kiliaridis S., Engstrdm C., Thilander B. (1985). The relationship between masticatory function and craniofacial morphology I. A cephalometric longitudinal analysis in the growing rat fed a soft diet. Eur. J. Orthod..

[38] Kiliaridis S. (1986). The relationship between masticatory function and craniofacial morphology III. The eruption pattern of the incisors in the growing rat fed a soft diet. Eur. J. Orthod..

[39] Kiliaridis S., Thilander B., Kjellberg H., Topouzelis N., Zafiriadis A. (1999). Effect of low masticatory function on condylar growth: A morphometric study in the rat. Am. J. Orthod. Dentofac. Orthop..

[40] Luca L., Roberto D., Francesca S.M., Francesca P. (2003). Consistency of diet and its effects on mandibular morphogenesis in the young rat. Prog. Orthod..

[41] Maki K., Nishioka T., Shioiri E., Takahashi T., Kimura M. (2002). Effects of dietary consistency on the mandible of rats at the growth stage: Computed X-ray densitometric and cephalometric analysis. Angle Orthod..

[42] Moore W.J. (1965). Masticatory function and skull growth. J. Zool..

[43] Ödman A., Mavropoulos A., Kiliaridis S. (2008). Do masticatory functional changes influence the mandibular morphology in adult rats. Arch. Oral Biol..

[44] Barber C.G., Green L.J., Cox G.J. (1963). Effects of the Physical Consistency of Diet on the Condylar Growth of the Rat Mandible. J. Dent. Res..

[45] Ulgen M., Baran S., Kaya H., Karadede I. (1997). The influence of the masticatory hypofunction on the craniofacial growth and development in rats. Am. J. Orthod. Dentofac. Orthop..

[46] Proffit W.R., Fields H.W., Sarver D.M. (2012). Contemporary orthodontic appliances. Contemporary Orthodontics.

[47] Moss M.L. (1997). The functional matrix hypothesis revisited. 1. The role of mechanotransduction. Am. J. Orthod. Dentofac. Orthop..

[48] Moss M.L. (1997). The functional matrix hypothesis revisited. 3. The genomic thesis. Am. J. Orthod. Dentofac. Orthop..

[49] Kiliaridis S., Mejersjö C., Thilander B. (1989). Muscle function and craniofacial morphology: A clinical study in patients with myotonic dystrophy. Eur. J. Orthod..

[50] Morel-Verdebout C., Botteron S., Kiliaridis S. (2007). Dentofacial characteristics of growing patients with Duchenne muscular dystrophy: A morphological study. Eur. J. Orthod..

[51] Egli F., Botteron S., Morel C., Kiliaridis S. (2018). Growing patients with Duchenne muscular dystrophy: Longitudinal changes in their dentofacial morphology and orofacial functional capacities. Eur. J. Orthod..

[52] Tsai C., Yang L., Chen K., Chiu W. (2010). The influence of masticatory hypofunction on developing rat craniofacial structure. Int. J. Oral Maxillofac. Surg..

[53] Rodrigues L., Traina A.A., Nakamai L.F., Luz J.G. (2009). Effects of the unilateral removal and dissection of the masseter muscle on the facial growth of young rats. Braz. Oral Res..

[54] Horowitz S.L., Shapiro H.H. (1955). Modification of skull and jaw architecture following removal of the masseter muscle in the rat. Am. J. Phys. Anthropol..

[55] Sengupta P. (2013). The Laboratory Rat: Relating Its Age with Human’s. Int. J. Prev. Med..

[56] Roach H.I., Mehta G., Oreffo R.O., Clarke N.M., Cooper C. (2003). Temporal Analysis of Rat Growth Plates: Cessation of Growth with Age Despite Presence of a Physis. J. Histochem. Cytochem..

[57] McCutcheon J.E., Marinelli M. (2009). Age matters. Eur. J. Neurosci..

[58] Arifin W.N., Zahiruddin W.M. (2017). Sample Size Calculation in Animal Studies Using Resource Equation Approach. Malays. J. Med. Sci..

[59] Charan J., Kantharia N.D. (2013). How to calculate sample size in animal studies?. J. Pharmacol. Pharmacother..

[60] Chia R., Achilli F., Festing M.F.W., Fisher E.M.C. (2005). The origins and uses of mouse outbred stocks. Nat. Genet..

[61] Athanasiou A.E. (1995). The Technique of Cephalometric Radiography. Orthodontic Cephalometry.

[62] Bulut E., Şahin B., Muğlalı M., Bekçioğlu B. (2012). Comparison of the planimetry and point-counting methods for the assessment of the size of the mandible cysts on orthopantomograms. Med. Oral Patol. Oral Cir. Bucal..

[63] Marcus L.F., Rohlf F.J., Bookstein F.L. (1990). Traditional morphometrics. Proceedings of the Michigan Morphometric Workshop.

[64] Geller S.A., Horowitz R.E. (2014). Gross examination. Methods Mol. Biol..

