# Supplementary material for: Impact of Diet Consistency on the Mandibular Morphology: A Systematic Review of Studies on Rat Models
Source: Int J Environ Res Public Health. 2022 Feb 25;19(5):2706. doi: 10.3390/ijerph19052706 (PMC8910531; doi:10.3390/ijerph19052706)
Supplement: Supplementary file 1 [file ijerph-19-02706-s001.zip › ijerph-1590889-supplementary.pdf]

Supplementary Table S1. Eligibility criteria for the present systematic review.

Domain	Inclusion Criteria	Exclusion Criteria
**Participants**	▪ Rats of any age, gender, strain that were fed with hard and soft food for at least 1 month.	▪ Other species than rats.
**Interventions**	▪ Rats that were divided into a hard diet group and a soft diet group for at least 1 month.	▪ Rats undergoing any kind of additional procedures in conjunction with the hard or soft diet.
**Comparisons**	▪ Hard food with soft food	
**Outcomes**	▪ Quantitative data regarding the effect of hard and soft food on morphological anatomy of the mandible measured in various ways (cephalometric analyses, morphometric analyses, gross morphological measurements, etc.)	▪ Qualitative data regarding the effect of hard and soft food on the morphological anatomy of the mandible.<br>▪ Histologic/histomorphometric measurements, measurements of immunohistochemistry<br>▪ Measurements based on drawings and sketches
**Study design**	▪ Experimental prospective controlled studies (according to the Scottish Intercollegiate Guidelines Network algorithm for classifying study design (available at http://www.sign.ac.uk/ assets/study_design.pdf (accessed on 1 January 2022)	▪ Non-comparative studies.<br>▪ *In vitro* or *exvivo* studies.<br>▪ Reviews, systematic reviews and meta-analyses.<br>▪ Fewer than 5 subjects per group analysed (Mead, 1988)

Supplementary Table S2. Strategy for database search (up to January 2022).

Database	Search Strategy	Hits
**General Sources**		
**PubMed**<br>http://www.ncbi.nlm.nih.gov/pubmed<br>(accessed on 1 January 2022)	((("Growth and Development"[Mesh]) AND "Diet"[Mesh]) AND "Rats"[Mesh])	**13,468**
	((("Rats"[Mesh]) AND "Diet"[Mesh]) AND "Mandible"[Mesh])	**81**
	(((("Growth and Development"[Mesh]) AND "Diet"[Mesh]) AND "Mandible"[Mesh]) AND "Rats"[Mesh])	**37**
	("food hardness" OR "food consistency" OR "soft diet" OR "hard diet" OR "soft food" OR "hard food" OR "diet consistency" OR "diet hardness" OR masticatory) AND (morphology OR mandib* OR ramus OR coronoid OR condyl* OR corpus) AND rat	**658**

**Scopus** https://www.scopus.com/search/form.url?zone=TopNavBarandorigin=searchbasic (accessed on 1 January 2022)	TITLE-ABS (("food hardness" OR "food consistency" OR "soft diet" OR "hard diet" OR "soft food" OR "hard food" OR "diet consistency" OR "diet hardness" OR masticatory) AND (morphology OR mandib* OR ramus OR coronoid OR condyl* OR corpus) AND rat)	**140**
**Web of Science™** http://apps.webofknowledge.com (accessed on 1 January 2022)	TITLE: (("food hardness" OR "food consistency" OR "soft diet" OR "hard diet" OR "soft food" OR "hard food" OR "diet consistency" OR "diet hardness" OR masticatory) AND (morphology OR mandib* OR ramus OR coronoid OR condyl* OR corpus) AND rat); Timespan: All years; Search language=Auto	**151**
**Regional sources**		
**Grey literature sources**		
**ProQuest Dissertations and Theses Global** http://search.proquest.com/dissertations (accessed on 1 January 2022)	ti(("food hardness" OR "food consistency" OR "soft diet" OR "hard diet" OR "soft food" OR "hard food" OR "diet consistency" OR "diet hardness" OR masticatory) AND (morphology OR mandib* OR ramus OR coronoid OR condyl* OR corpus) AND rat)[Full text]	**365**
